# Supplementary figures and images for: Administration of Bifidobacterium animalis subsp. lactis strain BB-12® in healthy children: characterization, functional composition, and metabolism of the gut microbiome
Source: Front Microbiol. 2023 May 12;14:1165771. doi: 10.3389/fmicb.2023.1165771 (PMC10275293; doi:10.3389/fmicb.2023.1165771)

*Day 30*

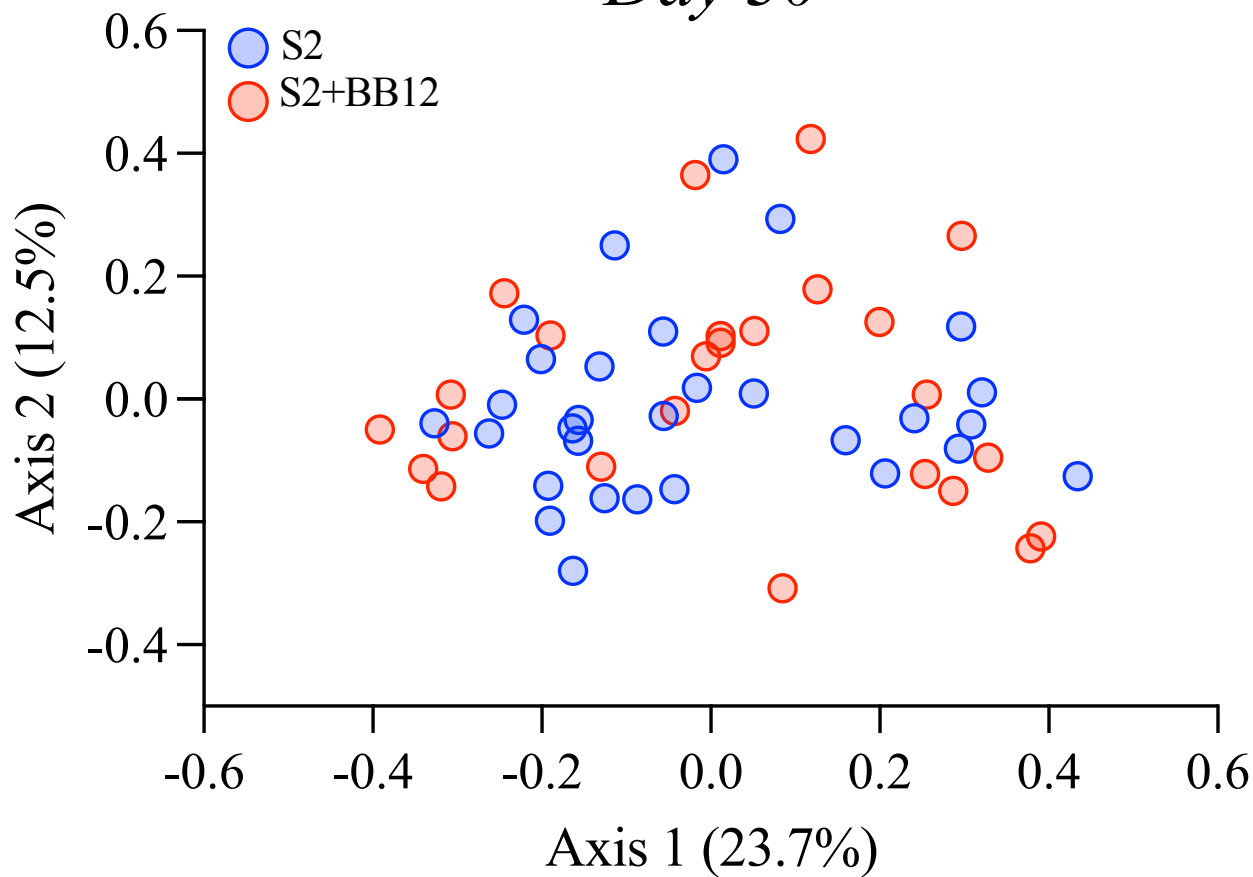

Supplement: SUPPLEMENTARY FIGURE S1 — Metagenomics PCoA day 30. [file Data_Sheet_1.PDF]

S2

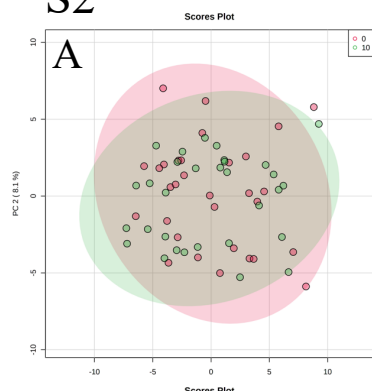

S2+BB12

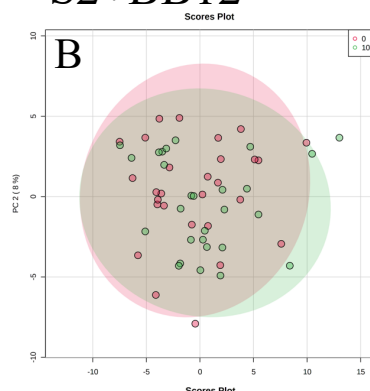

*Day 0 vs. Day 10*

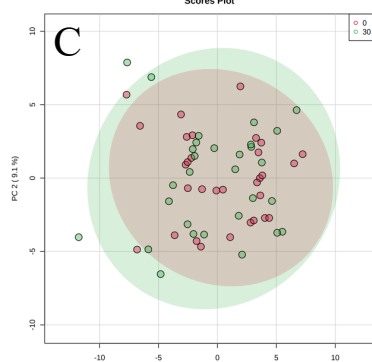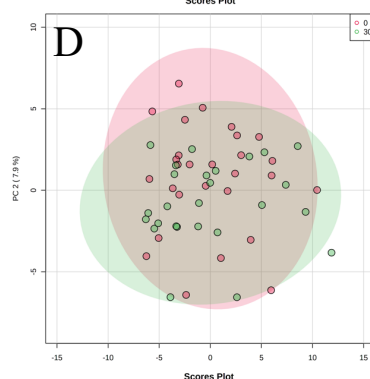

*Day 0 vs. Day 30*

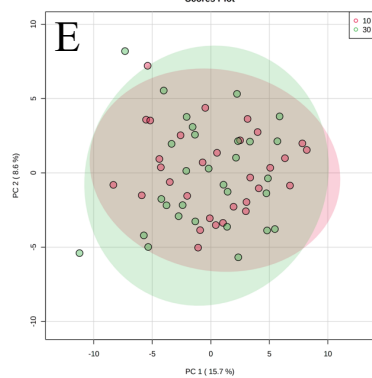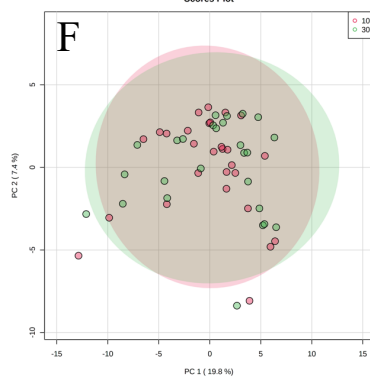

*Day 10 vs. Day 30*

Supplement: SUPPLEMENTARY FIGURE S2 — Metabolite PCA. [file Data_Sheet_2.PDF]
